# Study of Association of *CD40-CD154* Gene Polymorphisms with Disease Susceptibility and Cardiovascular Risk in Spanish Rheumatoid Arthritis Patients

**DOI:** 10.1371/journal.pone.0049214

**Published:** 2012-11-15

**Authors:** Mercedes García-Bermúdez, Carlos González-Juanatey, Raquel López-Mejías, María Teruel, Alfonso Corrales, José A. Miranda-Filloy, Santos Castañeda, Alejandro Balsa, Benjamín Fernández-Gutierrez, Isidoro González-Álvaro, Carmen Gómez-Vaquero, Ricardo Blanco, Javier Llorca, Javier Martín, Miguel A. González-Gay

**Affiliations:** 1 Instituto de Parasitología y Biomedicina López-Neyra, IPBLN-C.S.I.C., Granada, Spain; 2 Cardiology Division, Hospital Xeral-Calde, Lugo, Spain; 3 Department of Rheumatology, Hospital Universitario Marqués de Valdecilla, IFIMAV, Santander, Spain; 4 Department of Rheumatology, Hospital Xeral-Calde, Lugo, Spain; 5 Department of Rheumatology, Hospital Universitario La Princesa, IIS-Princesa, Madrid, Spain; 6 Rheumatology Unit, Hospital Universitario La Paz, Madrid, Spain; 7 Department of Rheumatology, Hospital Clinico San Carlos, Madrid, Spain; 8 Department of Rheumatology, Hospital Universitario de Bellvitge, IDIBELL, Barcelona, Spain; 9 Department of Epidemiology and Computational Biology, School of Medicine, University of Cantabria, and CIBER Epidemiología y Salud Pública (CIBERESP), IFIMAV, Santander, Spain; Centro Cardiologico Monzino IRCCS, Italy

## Abstract

**Objective:**

Rheumatoid arthritis (RA) is a chronic inflammatory disease associated with increased cardiovascular (CV) mortality. Since *CD40-CD154* binding has direct consequences on inflammation process initiation, we aimed to replicate previous findings related to disease susceptibility in Spanish RA population. Furthermore, as the major complication in RA disease patients is the development of CV events due to accelerated atherosclerosis, and elevated levels of CD40L/CD154 are present in patients with acute myocardial infarction, we assessed the potential association of *CD40* and *CD154/CD40L* gene variants with CV risk in Spanish RA patients.

**Methods:**

One thousand five hundred and seventy-five patients fulfilling the 1987 ACR classification criteria for RA and 1600 matched controls were genotyped for the *CD40* rs1883832, rs4810485 and rs1535045 and *CD154* rs3092952 and rs3092920 gene polymorphisms, using predesigned TaqMan single nucleotide polymorphism genotyping assays. Afterwards, we investigated the influence of *CD40-CD154* gene variants in the development of CV events. Also, in a subgroup of 273 patients without history of CV events, we assessed the influence of these polymorphisms in the risk of subclinical atherosclerosis determined by carotid ultrasonography.

**Results:**

Nominally significant differences in the allele frequencies for the rs1883832 ***CD40*** gene polymorphism between RA patients and controls were found (*p* = 0.038). Although we did not observe a significant association of *CD40-CD154* gene variants with the development of CV events, an ANCOVA model adjusted for sex, age at the time of the ultrasonography assessment, follow-up time, traditional CV risk factors and anti-cyclic citrullinated peptide antibodies disclosed a significant association (*p* = 0.0047) between *CD40* rs1535045 polymorphism and carotid intima media thickness, a surrogate marker of atherosclerosis.

**Conclusion:**

Data from our pilot study indicate a potential association of rs1883832 *CD40* gene polymorphism with susceptibility to RA. Also, the *CD40* rs1535045 gene variant may influence development of subclinical atherosclerosis in RA patients.

## Introduction

Rheumatoid arthritis (RA), the prototype of inflammatory chronic disease, is characterized by a consistent increase of cardiovascular (CV) mortality and morbidity, mainly due to a process of accelerated atherosclerosis [1], [2], [3]. There is some evidence supporting that both immune dysregulation and a systemic inflammatory milieu predating and characterizing earlier phases of RA may be important pathogenic factors of vascular impairment in patients with this condition. Interesting findings arise from the observation that CD40–CD40 ligand (CD40L/CD154) interaction, a crucial step in autoimmune disease pathogenesis, is thought to be involved in atherogenesis and plaque rupture in RA [4].

Once that CD40 interacts with its ligand (CD40L, aka CD154) on T cells, it acts as a transmembrane signal transducer leading to activation of intracellular kinases and transcription factors, giving rise to inflammatory responses [5]. CD40 signalling has been linked to pathogenic processes of chronic inflammatory and autoimmune diseases [6]. As a consequence of CD154/CD40L binding to CD40, release of cytokines and expression of adhesion molecules, as well as numerous inflammatory processes are triggered [7]. In addition, CD40/CD154 interactions may induce the expression of matrix metalloproteinases that degrade components of the atherosclerotic plaques and promote neovascularization, instability and plaque rupture leading to acute CV events or sudden death [8] and can play a prominent role in thrombotic events after plaque rupture [5]. This is relevant as the main cause of mortality and morbidity of RA is atherosclerosis, driving to CV events that are preceded by subclinical atherosclerosis.

CD40 is a 45–50 kDa membrane glycoprotein, member of the TNF-receptor superfamily, which acts as a receptor for CD154. *CD40* has been reported to be constitutively and/or inducibly expressed on B cells, platelets, monocytes and macrophages, endothelial cells, smooth muscle cells, mast cells, fibroblasts, dendritic cells, neutrophils and T cells [9] and also in the synovial fluid of RA patients [10].

A Genome Wide Association Study and meta-analysis highlighted the role of the major allele of the rs4810485 *CD40* polymorphism on chromosome 20q13 in the susceptibility to RA [11], [12]. Further replication studies in different populations [13], [14], [15] confirmed this association. Moreover, a recent report disclosed that the rs4810485 *CD40* variant may affect CD40 mRNA and protein expression on B cells and monocytes [16], and is in high linkage disequilibrium (*r* ^2^ = 0.95) with rs1883832, which has been shown to influence the efficiency of CD40 protein translation by disrupting a Kozak sequence [17], a stretch of nucleotides that flanks the start codon important for the initiation of translation of a nascent mRNA molecule [18]. Indeed, the major allele of rs1883832 increases the translational efficacy of CD40 transcripts, resulting in a 15–32% increase in CD40 protein production [19]. Furthermore, major alleles of rs4810485 and rs1883832 *CD40* genetic variants are associated with Grave's disease (GD) [17]; and also minor alleles have been associated with multiple sclerosis [20], [21], Crohn's disease [20] and giant cell arteritis [22], although with relatively weak statistical evidence.

CD154, also known as CD40L or TNFSF5, is a 39 kDa transmembrane protein with particular importance in T-cell dependent humoral immune responses. CD154 is expressed on various cell types, including cell types present in atherosclerotic plaques such as endothelial cells, monocytes, macrophages and smooth muscle cells [23]. Increased levels of soluble CD154 (sCD154) have been found in patients with systemic lupus erythematosus (SLE), RA, and Sjögren’s syndrome in association with disease activity [24]. Moreover, serum levels of sCD154 are higher in patients with RA than in healthy individuals and they correlate with both IgM-RF and IgG-RF titers [25]. Located in chromosome Xq26, *CD154* −3459A>G (rs3092952) variant has been reported to influence CD40L plasma levels, and those levels above median seem to reflect a prothrombotic state which can be managed with the use of antithrombotic treatment [26]. Also, rs3092920 *CD154* polymorphism is in high linkage disequilibrium (r^2^ = 0.97) with a microsatellite located in 3′-UTR that has been associated in Spanish population with SLE [27], another autoimmune disease associated with accelerated atherosclerosis and increased risk of CV complications, and with RA susceptibility [28]. sCD40L is associated with measures of subclinical atherosclerosis in the general population [8], and increased levels of sCD40L have been found in patients with acute myocardial infarction (MI) and unstable angina [7]. The role of the *CD40L* in the susceptibility to autoimmune diseases has not been investigated as broadly as the *CD40*, mainly due to this gene being located on the × chromosome. The different prevalence of some of autoimmune diseases between both genders [29] can suggest that genes located on the × chromosome could be susceptibility factors in these diseases; however, few studies analyzing polymorphisms on this chromosome has been made. Mutations on *CD40L* gene are associated with X-Linked Hyper-IgM syndrome; a family genetic disorder characterized by an increase of IgM level and a decrease of IgG and IgA [30]; other mutations and/or altered expression of *CD40L*, common γ chain, *FOXP3* (forkhead box P3) among other genes encoded on the × chromosome, are known to be cause of immune disorders such as X-SCID and IPEX dramatically manifested in men, while females can compensate or reduce severity of the symptoms due to the second × chromosome.

Taking together all these considerations, in the present study we aimed to confirm the role of *CD40* polymorphisms in the susceptibility to RA in Spanish population. In a second step, we assessed whether polymorphisms in *CD40-CD154* genes may influence the susceptibility to CV disease in RA. For this purpose, we analyzed the implication of these polymorphisms in the risk of CV events and presence of subclinical atherosclerosis in RA.

## Results and Discussion

Information on the main demographical data, clinical characteristics of the RA patients (n = 1575) enrolled in the current study, CV risk factors and CV events of patients is shown in **Table 1**. Two hundred and ninety (18.41%, 114 men and 176 women) of these 1575 RA patients experienced clinically evident CV events: ischemic heart disease, heart failure, cerebrovascular accident or peripheral arteriopathy.

**Table 1:** Clinical and demographic characteristics of rheumatoid arthritis (RA) patients included in the study, stratified by gender or by Cardiovascular events.

	Total	Males, N (%)	Females, N (%)	With CV events, N (%)	Without CV events, N (%)
RA Patients	1575	407 (25.84)	1168 (74.16)	290 (18.41)	1285 (81.59)
Age of patients at the time of disease diagnosis, years, median (IQR)	54 (43–64)	57 (46–68)	53 (42–63)	62.4 (53–73)	52 (41–61)
Time follow up, years, median (IQR)	10.8 (5.5–17)	9.7 (5.5–15)	11 (5.5–18)	11 (6–19)	10.59 (5–16.7)
Anti-CCP positive (n = 1285)	745 (57.98)	185 (57.99)	567 (58.69)	112 (52.83)	633 (58.99)
Rheumatoid Factor positive (n = 1548)	1078 (69.64)	281 (71.14)	797 (69.12)	188 (69.63)	890 (69.64)
Shared epitope, presence (n = 1230)	773 (62.85)	212 (64.05)	561 (62.40)	155 (64.05)	618 (62.55)
Cardiovascular events	290 (18.41)	114 (28.09)	176 (15.06)	290 (18.41)	-
Ischemic heart disease	152 (9.6)	74 (18.33)	78 (6.68)	152 (9.6)	-
Cerebrovascular accidents*	78 (4.97)	27 (6.73)	51 (4.36)	78 (4.97)	-
Heart failure	84 (5.5)	16 (4.07)	68 (6.02)	84 (5.5)	-
Peripheral arteriopathy	36 (2.36)	15 (3.81)	21 (1.85)	36 (2.36)	-
Hypertension (n = 1565)	540 (40.89)	169 (42.14)	471 (40.46)	202 (67.33)	438 (34.62)
Diabetes mellitus (n = 1560)	206 (13.21)	66 (16.58)	140 (12.05)	73 (24.41)	133 (10.51)
Dyslipidemia (n = 1561)	616 (39.46)	143 (35.57)	473 (40.81)	167 (55.30)	449 (35.66)
Obesity (n = 1480)	345 (23.31)	84 (22.52)	261 (23.58)	81 (29.14)	264 (21.96)
Smoking habit** (n = 1495)	425 (28.43)	171 (44.76)	254 (22.82)	94 (32.64)	331 (27.42)

Except where indicated otherwise, values are n (%). IQR: Interquartile Range. Anti-CCP: anti-Cyclic Citrullinated Peptide antibodies.

* A patient was considered to have a cerebrovascular accident when he/she had a stroke and/or transient ischemic attacks (TIAs). Strokes were classified according to their clinical features and they were confirmed by computed tomography and/or magnetic resonance imaging. TIAs were diagnosed if the symptoms were self-limited in less than 24 hours, without residual neurological damage [41].

** Smoking habit encompassed to those patients who smoked at the time of disease diagnosis, during the follow-up or who had smoked within the 10 years before the onset of RA symptoms or the disease diagnosis.

Patients with CV events were older at the time of disease diagnosis, were more likely to be men and more often had hypertension, diabetes mellitus, dyslipidemia and obesity. Moreover, smoking habit (defined as those patients who smoked at the time of disease diagnosis, during the follow-up or who had smoked within the 10 years before the onset of RA symptoms or the disease diagnosis) was more frequent in men than in women RA patients (44.76% *vs.* 22.82%). Epidemiological studies have shown that among environmental factors, cigarette smoking is one of the most relevant causes involved in RA pathogenesis [31], [32], [33], [34], [35]. It seems to play a relevant role in disease induction and progression, particularly in predisposed individuals [36]. On the other hand, a recent study has disclosed that circulating levels of sCD40L are increased in smokers during the early phase of acute MI [37], although number of patients involved was small. Also, although in some studies there was a synergist effect of smoking and genetic factors on the increased risk of cardiovascular disease of patients with rheumatoid arthritis, in our population we found no additive effect of smoking and *CD40-CD154* gene polymorphisms (data not shown).

Genotyping success rate was>95% overall. The estimated statistical power of the study to detect genetic modest effects like OR 1.17 in our cohort, was>75% for rs1883832, rs4810485 and rs1535045 *CD40* polymorphisms; and 80% (in women population) and 46% (men) for rs3092952 and 63% (women) and 35% (men) for rs3092920 to detect an OR = 1.25 in the case of *CD154* polymorphisms, assuming a prevalence of RA in Spanish population (0.5%) [38], under the condition of α error 0.05 and β error 0.20.

The statistical power of this study to detect a difference between absence or presence of CV disease in RA patients with an estimated OR 1.25, a type I error rate of 0.05, type II error rate of 0.20, and 0.05% of population risk, was 61% for *CD40* single nucleotide polymorphisms (SNPs) and between 48% (male) and 53% (female patients) for the rs3092952 polymorphism and between 34% (male) and 36% (female) for the rs3092920 *CD154* gene variant.

### Minor Allele Frequencies of the *CD40* rs1883832, rs4810485 and rs1535045, and CD154 rs3092952 and rs3092920 Polymorphisms in RA Patients and Controls

Genotype frequencies were conformed to Hardy-Weinberg equilibrium (p>0.05) both in patients and controls for all genetic variants under study.

A nominally significant difference in the allele frequency of the *CD40* rs1883832 gene polymorphism between RA patients and controls was seen (*p* = 0.038), supporting a role of this *CD40* 5′UTR variant in the susceptibility to RA in Spanish population (**Table 2**). On the other hand, *CD40* rs4810485 variant was marginally associated with disease susceptibility (*p* = 0.069). However, no association or trend was detected when allele or genotype frequencies of the *CD40* rs1535045 polymorphism in RA patients were compared with those observed in controls (**Table 2**). Allele frequencies observed for rs1883832 and rs4810485 *CD40* genetic polymorphisms (27.7% and 27.2%, respectively) were slightly different from those reported in HapMap CEU population (22.5% and 23%, respectively). The rs1535045 allele frequency (24.7%) was similar to the reported in HapMap CEU population (25%).

**Table 2:** Genotype and minor allele frequencies of SNPs located within *CD40* (20q13) and *CD40L* (Xq26) genes in RA Spanish patients and healthy controls.

					GENOTYPE, N (%)			ALLELE TEST		
Locus	Gene	SNP	1/2	Subgroup	1/1	1/2	2/2	MAF	*p*-value^a^	OR [95% CI]^b^
20q12	CD40	rs1883832	C/T	CONTROLS (n = 1545)	814 (52.69)	607 (39.29)	124 (8.02)	0.277		
				RA (n = 1510)	839 (55.56)	577 (38.21)	94 (6.23)	0.253	**0.038**	0.89 [0.79–0.99]^c^
				without CV events (n = 1227)	685 (55.83)	464 (37.82)	78 (6.36)	0.253		
				with CV events (n = 283)	154 (54.42)	113 (39.93)	16 (5.65)	0.256	0.86	1.02 [0.82–1.26]^d^
20q12	CD40	rs4810485	G/T	CONTROLS (n = 1548)	828 (53.49)	597 (38.57)	123 (7.95)	0.272		
				RA (n = 1503)	839 (55.82)	571 (37.99)	93 (6.19)	0.252	0.069	0.90 [0.80–1.01]^c^
				without CV events (n = 1219)	686 (56.28)	457 (37.49)	76 (6.23)	0.25		
				with CV events (n = 284)	153 (53.87)	114 (40.14)	17 (5.99)	0.26	0.59	1.06 [0.85–1.31]^d^
20q12	CD40	rs1535045	C/T	CONTROLS (n = 1558)	887 (56.93)	572 (36.71)	99 (6.35)	0.247		
				RA (n = 1519)	847 (55.76)	579 (38.12)	93 (6.12)	0.252	0.67	1.03 [0.91–1.15]^c^
				without CV events (n = 1236)	684 (55.34)	472 (38.19)	80 (6.47)	0.256		
				with CV events (n = 283)	163 (57.60)	107 (37.81)	13 (4.59)	0.235	0.31	0.89 [0.72–1.11]^d^
Xq26.3	CD154	rs3092952	A/G	Female CONTROLS (n = 890)	610 (68.5)	246 (27.6)	34 (3.8)	0.176		
				Females RA (n = 1115)	740 (66.37)	335 (30.04)	40 (3.59)	0.186	0.20	1.11 [0.94–1.31]^c^
				without CV events (n = 943)	621 (65.85)	286 (30.33)	36 (3.82)	0.189		
				with CV events (n = 172)	119 (69.19)	49 (28.49)	4 (2.33)	0.166	0.29	0.85 [0.62–1.16]^d^
				Male CONTROLS (n = 676)	568 (84.02)	–	108 (15.98)	0.160		
				Male RA (n = 375)	316 (84.27)	–	59 (15.73)	0.157	0.93	1.02 [0.71–1.46]^c^
				without CV events (n = 268)	226 (84.33)	–	42 (15.67)	0.157		
				with CV events (n = 107)	90 (84.11)	–	17 (15.89)	0.159	0.96	1.02 [0.52–1.95]^d^
Xq26.3	CD154	rs3092920	G/T	Female CONTROLS (n = 878)	707 (80.5)	156 (17.8)	15 (1.7)	0.106		
				Female RA (n = 1115)	892 (80.00)	209 (18.74)	14 (1.26)	0.106	0.97	1.00 [0.82–1.24]^c^
				without CV events (n = 940)	751 (79.89)	177 (18.83)	12 (1.28)	0.107		
				with CV events (n = 175)	141 (80.57)	32 (18.29)	2 (1.14)	0.103	0.82	0.96 [0.65–1.41]^d^
				Male CONTROLS (n = 669)	587 (87.74)	–	82 (12.26)	0.123		
				Male RA (n = 374)	339 (90.64)	–	35 (9.36)	0.094	0.16	0.74 [0.48–1.14]^c^
				without CV events (n = 269)	242 (89.96)	–	27 (10.04)	0.100		
				with CV events (n = 105)	97 (92.38)	–	8 (7.62)	0.076	0.47	0.74 [0.30–1.78]^d^

a *P*-value for the allelic model.

b Odds Ratio for the minor allele.

c with respect to Controls.

d comparing RA patients with CV events versus RA patients without CV events. MAF: minor allele frequency.

Because *CD154* is located on the × chromosome, and the elevated incidence of RA in women and higher frequency of atherosclerosis in men, we compared allele frequencies between patients and controls in males and females separately. No association was found between the *CD154* gene variants and susceptibility to RA disease (**Table 2**). Allele frequencies observed for rs3092952 and rs3092920 *CD154* gene variants (17.2% and 11.1%) were somewhat different from those reported in HapMap for CEU population (23% and 8%, respectively).

### *CD40* and *CD154* Variants and Risk of CV Events in Patients with RA

**Table 2** shows the genotype frequencies of the *CD40* rs1883832, rs4810485 and rs1535045 gene polymorphisms in this series of RA patients stratified according to the presence or absence of CV events. No association was found between *CD40* polymorphisms and CV events risk in our RA patients cohort. It was also the case for *CD154* gene variants in women or men RA patients.

Logistic regression model to determine the presence of CV disease in RA patients according to *CD40* rs1883832, rs4810485 and rs1535045 allele distribution did not disclose statistically significant differences between patients who suffer CV events against the ones who did not (**Table S1**). The same model was applied for the *CD154* variants, stratifying patients by gender, and no association was detected (**Table S2**). Analyses were adjusted for sex, age at RA diagnosis, follow-up time and presence or absence of shared epitope, hypertension, diabetes mellitus, dyslipidemia, obesity and smoking habit as confounder factors. In addition, we specifically assessed the influence of the variants in the occurrence of cardiac ischemic events, heart failure or cerebrovascular accidents. However, no significant association was found (data not shown), despite the fact that *CD154* −3459A>G (rs3092952) variant has been reported to influence CD40L plasma levels, although the −3459 G allele did not confer an increased risk of MI or mortality in the general population [26]. However, the CD40/CD154 system is involved in the increased inflammatory response and extracellular matrix degradation [39], leading to disruption of atherosclerotic plaque [40].

In a further step, to assess the independency of the polymorphisms in their association with clinically evident CV disease, we performed a conditional logistic regression analysis. However, no significant association was observed for them (**Tables S3** and **S4**), although a trend of association was found between *CD154* rs3092952 and rs3092920 variants in women after correction of the conditional analysis by age at RA diagnosis, follow-up time and presence or absence of shared epitope, and traditional cardiovascular risk factors as confounders.

No epistatic interactions of the *CD40* polymorphisms with *HLA-DRB1*-shared epitope [41] were observed (data not shown). Likewise, no significant associations were detected when we analyzed our cohort of patients by presence or not of hypertension, diabetes mellitus, dyslipidemia or anti-Cyclic Citrullinated Peptide antibodies (anti-CCP) status (data not shown).

We also analyzed the combined influence of the *CD40* rs1883832 and rs1535045 variants in the risk of CV disease comparing the frequency of their estimated haplotypes, but no significant differences were observed among combinations, before or after adjustment by sex, age, follow-up time or long-established CV risk factors (**Table S5**).

In line with the above, the analysis of the combined influence for *CD40L* rs3092952 and rs3092920 polymorphisms did not disclose association between allelic combinations and risk of CV events. Only three combinations were considered in males; a fourth combination was excluded from the analysis due to its low frequency: 1.56% among the subjects without CV disease and 0% in those with CV disease (**Table S5**).

### *CD40* rs1883832, rs4810485 and rs1535045 and *CD154* rs3092952 and rs3092920 Gene Polymorphisms and Subclinical Atherosclerosis

Since carotid intima-media thickness (IMT) has been found to predict the risk of CV events in the extended follow-up of patients with RA [42], in a further step we analyzed potential differences in the carotid IMT in 273 RA patients with no history of CV events stratified according to the genotype and allele distribution. In this regard, when we assessed the genotype distribution of the *CD40* rs1535045 polymorphism, we observed that RA patients carrying the CC *CD40* rs1535045 genotype (n = 155) had higher carotid IMT (0.75±0.19 mm) than those CT *CD40* rs1535045 heterozygous (n = 104; carotid IMT: 0.72±0.16 mm) or the homozygous for the minor T allele *CD40* rs1535045 (n = 14; carotid IMT: 0.70±0.16 mm). However, these differences in the genotype distribution did not reach statistical significance. In keeping with this observation when we assessed allele distribution of the *CD40* rs1535045 polymorphism, we observed that RA patients carrying the C allele of the *CD40* rs1535045 gene variant (n = 414) had higher carotid IMT (0.74±0.18 mm) than those carrying the less common T allele (n = 132; carotid IMT: 0.71±0.16 mm). Nevertheless, differences were not statistically significant (p = 0.13) (**Table 3**). It was also the case for the remaining *CD40* gene polymorphisms (**Table 3**), as well as *CD154* gene variants assessed in the study (**Table 4**).

**Table 3:** Comparison of carotid artery intima-media thickness (cIMT) according to *CD40* rs1883832, rs4810485 and rs1535045 polymorphisms.

rs1883832	cIMT mm,mean (SD)	*p*	rs4810485	cIMT mm,mean (SD)	*p*	rs1535045	cIMT mm,mean (SD)	*p*
CC (n = 143)	0.73 (0.17)		GG (n = 143)	0.73 (0.18)		CC (n = 155)	0.75 (0.19)	
CT (n = 107)	0.74 (0.18)		GT (n = 106)	0.74 (0.18)		CT (n = 104)	0.72 (0.16)	
TT (n = 23)	0.75 (0.16)		TT (n = 24)	0.75 (0.16)		TT (n = 14)	0.70 (0.16)	
Model		0.71			0.82	Model		0.29
C (n = 393)	0.73 (0.18)		G (n = 392)	0.73 (0.18)		C (n = 414)	0.74 (0.18)	
T (n = 153)	0.74 (0.17)	0.41	T (n = 154)	0.74 (0.17)	0.52	T (n = 132)	0.71 (0.16)	0.13

**Table 4:** Comparison of carotid artery intima-media thickness (cIMT) according to *CD154* rs3092920 and rs3092952 SNPs.

Gender	rs3092952	cIMTmean(SD)	*p*	rs3092920	cIMTmean(SD)	*p*
Women	AA (n = 130)	0.73(0.17)		GG (n = 161)	0.72(0.17)	
	AG (n = 69)	0.70(0.14)		GT (n = 37)	0.70(0.14)	
	GG (n = 4)	0.80(0.20)		TT (n = 3)	0.67(0.02)	
	Model		0.43			0.65
	A (n = 329)	0.72(0.16)		G (n = 359)	0.72(0.16)	
	G (n = 77)	0.71(0.15)	0.73	T (n = 43)	0.70(0.13)	0.34
Men	A (n = 57)	0.78(0.21)		G (n = 64)	0.78(0.21)	
	G (n = 9)	0.79(0.24)	0.91	T (n = 4)	0.71(0.09)	0.46

Interestingly, when the analysis of variance (ANOVA) model was adjusted for sex, age at the time of the ultrasonography assessment, follow-up time, and traditional CV risk factors (ANCOVA model), we did observe nominally significant evidence of association between carotid IMT and rs1535045 *CD40* polymorphism (*p* = 0.023) (**Table 5**); therefore, we carried out a permutation test with 100000 replications in order to control type I error; in the permutation test, the relationship between carotid IMT and rs1535045 *CD40* gene variant had *p* value = 0.048 (estimated with precision lower than 0.001). This result could be related to the fact that increased CD40 expression has been established in unstable atherosclerotic plaques [9], and carotid IMT is one of the parameters well known as a marker of early atherosclerosis. In addition, *CD40* rs1535045 SNP has previously been reported to be significantly associated with coronary artery calcification, which correlates with atherosclerosis and CV disease [39], although the SNP was not previously associated with carotid IMT. Since presence of anti-CCP antibodies had been previously associated with stronger evidence of CV disease in patients with RA [43], in a further step the ANCOVA model was also adjusted for anti-CCP status, that confirmed the statistically significant association between rs1535045 *CD40* polymorphism and carotid IMT (*p* = 0.0047). Conditional analysis of the rs1883832 and rs1535045 *CD40* gene variants in the adjusted ANCOVA model, rendered following *p*-values: 0.82 and 0.0047 for rs1883832 and rs1535045, respectively. No association was found in case of *CD154/CD40L* gene variants (**Table 6**). Our results indicate that a potential association between surrogate markers of atherosclerosis, in this case carotid IMT, and *CD40* rs1535045 polymorphism may exist. It may have clinical significance as carotid IMT has proved to be a good predictor of CV events in patients with RA [42]. Association between endothelial dysfunction, another surrogate marker of atherosclerosis that is considered an early step in the atherogenesis process, and other genetic markers has been reported by other studies in RA patients [44], [45], [46]. These findings showing association between markers of subclinical atherosclerosis and genetic polymorphisms in RA have clinical relevance as they can help to identify patients at risk before CV events may occur.

**Table 5:** Comparison of carotid intima-media thickness (cIMT) according to *CD40* rs1883832, rs4810485 and rs1535045 alleles in an adjusted ANCOVA model.

	cIMT
*CD40* rs1883832, *p* T vs. C*	0.62
*CD40* rs4810485, *p* T vs. G*	0.63
*CD40* rs1535045, *p* T vs. C*	**0.023**
*CD40* rs1883832, *p* T vs. C** †	0.59
*CD40* rs1535045, *p* T vs. C** †	**0.023**

* Analyses adjusted for sex, age at rheumatoid arthritis diagnosis, follow-up time, and traditional cardiovascular risk factors.

** Conditional analysis of the rs1883832 and rs1535045 *CD40* gene variants.

† Analyses adjusted for sex, age at rheumatoid arthritis diagnosis, follow-up time, traditional cardiovascular risk factors and anti-CCP status: *p* = 0.82 and 0.0047 for rs1883832 and rs1535045, respectively.

**Table 6:** Comparison of cIMT according to *CD154* rs3092952 and rs3092920 alleles in an adjusted ANCOVA model, stratified by gender.

Gender		cIMT
Women	*CD154* rs3092952, *p* G vs. A*	0.62
	*CD154* rs3092920, *p* T vs. G*	0.89
	*CD154* rs3092952, *p* G vs. A**	0.30
	*CD154* rs3092920, *p* T vs. G**	0.47
Men	*CD154* rs3092952, *p* G vs. A*	0.91
	*CD154* rs3092920, *p* T vs. G*	0.28
	*CD154* rs3092952, *p* G vs. A**	0.96
	*CD154* rs3092920, *p* T vs. G**	0.32

* Analyses adjusted for age at rheumatoid arthritis diagnosis, follow-up, and classic CV risk factors and anti-CCP status.

** Conditional analysis of the rs3092952 and rs3092920 *CD154* gene variants.

Looking for a relation between rs1535045 *CD40* gene variant and the presence of carotid plaques in patients with carotid IMT>1.0 mm, we did not find any significant association nor in the crude (allelic *p* = 0.71, 95% CI [0.23–1.81]) or adjusted by sex, age at RA diagnosis, follow-up time and traditional CV factors, model (allelic *p* = 0.65, 95% CI [0.23–1.83]), although number of patients involved was small (n = 19).

As in many other human genetic studies, potential limitations may exist in our study. In this regard, the phenotypes involved in the atherosclerosis disease, which some consider an inflammatory autoimmune disease [47], are complex and involve multiple small-to modest effects of multiple genes and environmental factors. Since increased carotid IMT is a surrogate marker of atherosclerosis that may allow identifying high risk patients in a subclinical phase of the disease, longer duration of follow-up may be required to see the clinical relevance of the *CD40* rs1535045 in the atherosclerosis disease of patients with RA. In keeping with our data showing association of subclinical atherosclerosis with *CD40* rs1535045 gene variant, a recent study on 681 Swedish patients with RA disclosed an association between stroke/transient ischemic accident in men with RA that were homozygous for the *CD40* rs1535045 polymorphism major allele [48]. Regrettably, although our study encompassed a larger number of patients, we could not confirm association of any of the *CD40* gene polymorphisms with clinically evident CV events in Spanish RA patients. There are a number of potential explanations that might enlighten these differences. First, RA is a complex disease and both the genetic backgrounds as well as environmental factors have clearly been shown to influence the phenotype expression of the disease and its potential complications. Second, the Swedish study included patients with longer follow-up (mean 15.5 years) from disease diagnosis than our cohort (median 10.8 years). Since the carotid IMT has been found to predict the development of CV events in the extended follow-up of patients with RA [42], a longer follow-up might be required to disclose a clinical association between rs1535045 polymorphism in *CD40* and CV events in our population.

In conclusion, our data may indicate a potential association of the single nucleotide polymorphism located in the 5′-UTR of the *CD40* gene (rs1883832, −1C/T) with susceptibility to RA in the Spanish population. Also, based on our results, a potential influence of the *CD40* rs1535045 variant in the development of subclinical atherosclerosis in RA patients may exist. However, given that no other independent cohort was available for replication; our study should be regarded as a pilot one. Therefore, further studies in population with different genetic backgrounds are required to confirm the implication of CD40/CD154 system in the atherosclerosis process associated to RA.

## Materials and Methods

### Patients and Controls

Between March 1996 and September 2008,1575 consecutive patients that fulfilled the 1987 American College of Rheumatology classification criteria for RA [49] were recruited from the Rheumatology Outpatient Clinics of Hospital Xeral-Calde (Lugo), Hospital Clínico San Carlos (Madrid), Hospital Universitario La Paz (Madrid), Hospital Universitario La Princesa (Madrid), Hospital Universitario Marqués de Valdecilla (Santander), and Hospital Universitario de Bellvitge (Barcelona), Spain. Patients and 1600 bone marrow and blood donors from National Repository DNA Bank (University of Salamanca, Spain), matched by age, sex and ethnicity, from the corresponding regions, were assessed for differences in the *CD40* rs1883832, rs4810485 and rs1535045, and *CD154* rs3092952 and rs3092920 gene variants.

### Study Protocol

#### Ethics Statement

A subject’s written consent was obtained according to the declaration of Helsinki, and propose of the work was approved by the Ethics Committee of Galicia (Spain). The Ethics Committees of the Hospital Clínico San Carlos (Madrid), Hospital La Paz (Madrid), Hospital de La Princesa (Madrid), Hospital Universitario Bellvitge (Barcelona) and Hospital Universitario Marqués de Valdecilla (Santander) also approved the study.

Between December 2009 and January 2010 patient’s clinical records were examined until patient’s death, loss of follow-up or December 1^st^, 2009. Information on the main demographic characteristics, CV risk factors and CV events of RA patients is shown in **Table 1**. Clinical definitions for CV events and classic CV risk factors were described elsewhere [41], [42], [50]. A CV event was considered to be present if the patient had ischemic heart disease, heart failure, cerebrovascular accident or peripheral arteriopathy.

DNA was obtained from patientś peripheral blood, using standard methods.

### Genotyping

#### *CD40* and *CD154* genotyping

Subjects were genotyped to determine:

*Three SNPs of *CD40* associated with other autoimmune diseases: rs1883832 located in the Kozak consensus sequence of the 5′-UTR, had been associated with GD [19]; while rs4810485, located in the first intron of the gene and in high linkage disequilibrium with rs1883832 (r^2^ = 0.95), has been identified as a risk factor for RA [11]. Moreover, the major allele of rs1535045 SNP, located in the first intron of the gene, has been associated with subclinical atherosclerosis in diabetes families [39].

*Two genetic variants located in 5′ UTR (rs3092952) and 3′ UTR (rs3092920) of *CD40L* (r^2^ = 0.38) were selected. These SNPs are located in different haplotype blocks of *CD40L* [51]. The variant rs3092920 is located near to 3′-UTR microsatellite, which was previously associated with RA and SLE [27], [28]; while rs3092952 is a functional variant related with the levels of sCD40LG in plasma [26].

*CD40* and *CD154* variants genotyping were made using TaqMan Assays-on-Demand and TaqMan Genotyping Master Mix, and analyzed using the ABI 7900HT Fast Real-Time PCR System (Applied Biosystems, Foster City, CA, USA), following manufacturer instructions. Negative controls and duplicates were included to check the accuracy of the genotyping.

#### Shared Epitope determination

Several *HLA-DRB1* alleles (HLA-DRB1*0401, *0404, *0405, *0408, *0101, *0102, *1001, *1402) are associated with susceptibility to RA. These alleles encode a conserved amino acid sequence called the shared epitope, at position 70–74 in the third hypervariable region of the HLA-DRβ1 molecule [52]. *HLA-DRB1* typing was carried out using a reverse dot-blot kit with sequence-specific oligonucleotide (SSO) probes (Dynal RELITM SSO HLA-DRB1 typing kit; Dynal Biotech, Bromborough, UK).

### Assessment of Carotid Plaque and Carotid Intima Media Thickness (IMT)

To determine the potential association between the *CD40/CD154 (CD40L)* polymorphisms and the presence of subclinical atherosclerosis, between March 2007 and September 2010 a random subgroup of patients (n = 273) with no previous history of CV events was assessed by carotid ultrasonography to determine the carotid IMT and carotid plaques [53], [54]. The common carotid arteries (CCAs) were evaluated with high resolution B-mode ultrasound (SONOS5500 and IE33, Philips Medical Systems, Andover, MA, USA) using a linear array 11-MHz probe with a standardized setup. Carotid IMT was measured at the posterior wall of the right and left CCAs, 10 mm from the carotid bifurcation, over the proximal 10-mm-long segment. The patients were placed in the supine position with their heads slightly bent in the opposite direction from the examination side. The right and left CCAs was first identified in B-mode, in a transverse view and followed from the proximal part to the bulb origin. Immediately afterwards, the CCAs and the most proximal part of the bulb were imaged in a longitudinal view from a lateral approach. Both common carotid arteries were scanned longitudinally to visualize the intima-media complex of the far wall of the artery. The segments of the CCAs 10 mm proximal to the carotid bifurcation were scanned. The image was focused on the posterior carotid wall in longitudinal view. For measurement of the IMT, the distance between the leading edges of the lumen-intima interface and the media-adventitia interface of the B-mode frame was taken. The IMT was calculated for both the left and the right CCA, and the CCA-IMT was defined as the maximum of these. An ECG recording during the ultrasound examination was obtained for all patients. All measurements were performed in the end-diastole. Carotid plaque was considered to be present when there was a localized irregular thickening of at least 1.5 mm.

### Statistical Analysis

All genotype data were checked for deviation from Hardy-Weinberg equilibrium (HWE) using http://ihg.gsf.de/cgi-bin/hw/hwa1.pl.

Comparison of proportions was carried out using χ^2^ test or Fisher test, when required. Strength of associations between CV events and genotypes or alleles of *CD40* and *CD154* variants were estimated using odds ratios (OR) and 95% confidence intervals (CI), via multiple logistic regression; estimates were further adjusted by sex, age at RA diagnosis, time of follow-up, presence or absence of the rheumatoid shared epitope, and classic CV risk factors (hypertension, diabetes mellitus, dyslipidemia, obesity and smoking habit) as potential confounders. Given *CD40LG* is located on the X-chromosome and the sex bias of RA disease, we performed the analysis separately to each gender for those gene variants.

The association between genotypes of the *CD40* and *CD154* SNPs and carotid IMT as surrogate marker of subclinical atherosclerosis were tested using unpaired t test to compare between 2 groups, and one-way analysis of variance (ANOVA) to compare among more than two groups. Moreover, we also tested the association between these parameters and alleles using analysis of covariance (ANCOVA) adjusting for gender, age and duration of the disease at the time of the ultrasonographic study, traditional CV risk factors, and anti-CCP status.

Nominal statistical significance was defined as p<0.05. Statistical significance, after applying a Bonferroni adjustment for 4 independent SNPs, was p<0.0125. We carried out a permutation test with 100,000 replications in order to control type I error, using the programme Stata 12/SE (StataCorp, College Station, TX, USA).

Statistical power for the study was calculated using “CaTS - Power Calculator for Two Stage Association Studies” (http://www.sph.umich.edu/csg/abecasis/CaTS/) [55].

## Supporting Information

Table S1 Logistic regression model to explain the presence of CV disease in patients with RA according to *CD40* rs1883832, rs4810485 and rs1535045 allele distribution.
(DOC)

Table S2 Logistic regression model to explain the presence of CV disease in RA patients stratified by gender according to *CD154* rs3092952 and rs3092920 allele distribution.
(DOC)

Table S3 Conditional logistic regression analysis of *CD40* rs1883832 and rs1535045 polymorphisms in the risk of cardiovascular disease in RA patients.
(DOC)

Table S4 Conditional logistic regression analysis of *CD154* rs3092952 and rs3092920 variants in CV disease risk stratified by gender (Xq26).
(DOC)

Table S5 Distribution of haplotypes of *CD40* and *CD154* gene variants in RA patients with and without CV disease.
(DOC)
