# Supplementary material for: Study of Association of CD40-CD154 Gene Polymorphisms with Disease Susceptibility and Cardiovascular Risk in Spanish Rheumatoid Arthritis Patients
Source: PLoS One. 2012 Nov 15;7(11):e49214. doi: 10.1371/journal.pone.0049214 (PMC3499567; doi:10.1371/journal.pone.0049214)
Supplement: Table S1 — Logistic regression model to explain the presence of CV disease in patients with RA according to CD40 rs1883832, rs4810485 and rs1535045 allele distribution. (DOC) [file pone.0049214.s001.doc]

**Suppl. Table S1.** Logistic regression model to explain the presence of CV disease in patients with RA according to *CD40* rs1883832, rs4810485 and rs1535045 allele distribution.

|  | *p* | OR [95% CI] | *p** | OR [95% CI]* |
| --- | --- | --- | --- | --- |
| rs1883832 T vs. C | 0.62 | 1.05 [0.86-1.28] | 0.53 | 1.09 [0.82-1.44] |
| rs4810485 T vs. G | 0.40 | 1.09 [0.89-1.33] | 0.26 | 1.17 [0.89-1.54] |
| rs1535045 T vs. C | 0.12 | 0.85 [0.69-1.04] | 0.24 | 0.84 [0.63-1.12] |

*Analyses adjusted for sex, age at rheumatoid arthritis diagnosis, follow-up time from the disease diagnosis, presence or absence of shared epitope, and traditional CV risk factors (hypertension, diabetes mellitus, dyslipidemia, obesity and smoking habit). OR [95% CI]: Odds Ratio with 95% Confidence Interval.
