# Supplementary material for: Study of Association of CD40-CD154 Gene Polymorphisms with Disease Susceptibility and Cardiovascular Risk in Spanish Rheumatoid Arthritis Patients
Source: PLoS One. 2012 Nov 15;7(11):e49214. doi: 10.1371/journal.pone.0049214 (PMC3499567; doi:10.1371/journal.pone.0049214)
Supplement: Table S2 — Logistic regression model to explain the presence of CV disease in RA patients stratified by gender according to CD154 rs3092952 and rs3092920 allele distribution. (DOC) [file pone.0049214.s002.doc]

**Suppl. Table S2.** Logistic regression model to explain the presence of CV disease in RA patients stratified by gender according to *CD154* rs3092952 and rs3092920 allele distribution.

| Gender |  | *p* | OR [95% CI] | *p** | OR [95% CI]* |
| --- | --- | --- | --- | --- | --- |
| Female | rs3092952, G vs. A | 0.29 | 0.85 [0.62-1.15] | 0.35 | 0.82 [0.54-1.24] |
|  | rs3092920, T vs. G | 0.82 | 0.96 [0.66-1.39] | 0.38 | 1.26 [0.75-2.12] |
| Male | rs3092952, G vs. A | 0.96 | 0.98 [0.53-1.82] | 0.65 | 0.81 [0.34-1.96] |
|  | rs3092920, T vs. G | 0.43 | 0.72 [0.32-1.64] | 0.53 | 0.67 [0.19-2.36] |

*Analyses adjusted for age at rheumatoid arthritis diagnosis, follow-up time from the disease diagnosis, presence or absence of shared epitope, and traditional CV risk factors. OR [95% CI]: Odds Ratio with 95% Confidence Interval.
