# Supplementary material for: Study of Association of CD40-CD154 Gene Polymorphisms with Disease Susceptibility and Cardiovascular Risk in Spanish Rheumatoid Arthritis Patients
Source: PLoS One. 2012 Nov 15;7(11):e49214. doi: 10.1371/journal.pone.0049214 (PMC3499567; doi:10.1371/journal.pone.0049214)
Supplement: Table S3 — Conditional logistic regression analysis of CD40 rs1883832 and rs1535045 polymorphisms in the risk of cardiovascular disease in RA patients. (DOC) [file pone.0049214.s003.doc]

**Suppl. Table S3**. Conditional logistic regression analysis of *CD40* rs1883832 and rs1535045 polymorphisms in the risk of cardiovascular disease in RA patients**.**

| SNP | *p* | OR [95% CI] | *p** | OR [95% CI]* |
| --- | --- | --- | --- | --- |
| rs1883832 | 0.52 | 1.07 [0.88-1.31] | 0.50 | 1.10 [0.83-1.45] |
| rs1535045 | 0.12 | 0.85 [0.67-1.04] | 0.22 | 0.84 [0.63-1.11] |

Results for rs4810485 gene variant are not shown since it is in high linkage disequilibrium (*r*^2^=0.95) with rs1883832.

*Analyses adjusted for gender, age at rheumatoid arthritis diagnosis, follow-up time from the disease diagnosis, presence or absence of shared epitope, and classic CV risk factors (hypertension, diabetes mellitus, dyslipidemia, obesity and smoking habit). OR [95% CI]: Odds Ratio with 95% Confidence Interval.
