# Supplementary material for: Study of Association of CD40-CD154 Gene Polymorphisms with Disease Susceptibility and Cardiovascular Risk in Spanish Rheumatoid Arthritis Patients
Source: PLoS One. 2012 Nov 15;7(11):e49214. doi: 10.1371/journal.pone.0049214 (PMC3499567; doi:10.1371/journal.pone.0049214)
Supplement: Table S4 — Conditional logistic regression analysis of CD154 rs3092952 and rs3092920 variants in CV disease risk stratified by gender (Xq26). (DOC) [file pone.0049214.s004.doc]

**Suppl. Table S4. .** Conditional logistic regression analysis of *CD154* rs3092952 and rs3092920 variants in CV disease risk stratified by gender (Xq26).

| Gender | SNP | *p* | OR [95% CI] | *p** | OR [95% CI]* |
| --- | --- | --- | --- | --- | --- |
| Female | rs3092952 | 0.25 | 0.80 [0.53-1.18] | 0.071 | 0.62 [0.37-1.04] |
|  | rs3092920 | 0.60 | 1.14 [0.70-1.86] | 0.068 | 1.83 [0.96-3.50] |
| Male | rs3092952 | 0.96 | 0.98 [0.42-2.31] | 0.62 | 0.75 [0.23-2.43] |
|  | rs3092920 | 0.61 | 0.75 [0.26-2.21] | 0.92 | 0.92 [0.18-4.71] |

*Analyses adjusted by age at rheumatoid arthritis diagnosis, follow-up time and presence or absence of shared epitope, and traditional cardiovascular factors (hypertension, diabetes mellitus, dyslipidemia, obesity and smoking habit). OR [95% CI]: Odds Ratio with 95% Confidence Interval.
