# Supplementary material for: Study of Association of CD40-CD154 Gene Polymorphisms with Disease Susceptibility and Cardiovascular Risk in Spanish Rheumatoid Arthritis Patients
Source: PLoS One. 2012 Nov 15;7(11):e49214. doi: 10.1371/journal.pone.0049214 (PMC3499567; doi:10.1371/journal.pone.0049214)
Supplement: Table S5 — Distribution of haplotypes of CD40 and CD154 gene variants in RA patients with and without CV disease. (DOC) [file pone.0049214.s005.doc]

**Suppl. Table S5.** Distribution of haplotypes of *CD40* and *CD154* gene variants in RA patients with and without CV disease.

| Gender | *CD40* rs1883832-rs1535045 | With CV events, n (%) | Without CV events, n (%) | *p* | OR [95% CI] | *p^a^* | OR [95% CI]^a^ |
| --- | --- | --- | --- | --- | --- | --- | --- |
| Male+Female | C-C | 351 (57.35) | 1331 (55.83) | - | Ref. | - | Ref. |
|  | T-C | 121 (19.77) | 435 (18.25) | 0.65 | 1.05 [0.84-1.33] | 0.33 | 1.17 [0.85-1.61] |
|  | C-T | 100 (16.34) | 455 (19.09) | 0.15 | 0.83 [0.65-1.07] | 0.53 | 0.90 [0.64-1.25] |
|  | T-T | 40 (6.54) | 163 (6.84) | 0.70 | 0.93 [0.65-1.34] | 0.44 | 0.82 [0.50-1.35] |
| Gender | *CD154* rs3092952-rs3092920^a^ | With CV events, n (%) | Without CV events, n (%) | *p* | OR [95% CI] | *p^a^* | OR [95% CI]^a^ |
| Female | A-G | 279 (81.58) | 1479 (79.86) | - | Ref. | - | Ref. |
|  | G-T | 29 (8.48) | 178 (9.61) | 0.49 | 0.86 [0.57-1.30] | 0.72 | 1.11 [0.63-1.95 |
|  | G-G | 28 (8.19) | 175 (9.45) | 0.44 | 0.85 [0.56-1.29] | 0.12 | 0.65 [0.38-1.12] |
|  | A-T | 6 (1.75) | 20 (1.08) | 0.32 | 1.59 [0.63-3.99] | 0.20 | 2.22 [0.65-7.60] |
| Male | A-G | 90 (86.54) | 213 (82.88) | - | Ref. | - | Ref. |
|  | G-T | 8 (7.69) | 22 (8.56) | 0.73 | 0.86 [0.37-2.01] | 0.63 | 0.73 [0.20-2.64] |
|  | G-G | 6 (5.77) | 18 (7.00) | 0.63 | 0.79 [0.30-2.05] | 0.57 | 0.70 [0.21-2.37] |

^a^Analyses adjusted for gender, age at rheumatoid arthritis diagnosis, follow-up time, presence or absence of share epitope, and classic CV risk factors. ^b^According to gender stratification. Only three haplotypes were considered in males; a fourth combination was excluded from the analysis due to its low frequency: 1.56% among the subjects without CV disease and 0% in those with CV disease.
